# Effect of Temperature Shock and Inventory Surprises on Natural Gas and Heating Oil Futures Returns

**DOI:** 10.1155/2014/457636

**Published:** 2014-07-13

**Authors:** John Wei-Shan Hu, Yi-Chung Hu, Chien-Yu Lin

**Affiliations:** ^1^Department of Business Administration, Chung Yuan Christian University, Chung Li 32023, Taiwan; ^2^Department of Finance, Chung Yuan Christian University, Chung Li 32023, Taiwan; ^3^Huang Lin Co., Taoyuan 33742, Taiwan

## Abstract

The aim of this paper is to examine the impact of temperature shock on both near-month and far-month natural gas and heating oil futures returns by extending the weather and storage models of the previous study. Several notable findings from the empirical studies are presented. First, the expected temperature shock significantly and positively affects both the near-month and far-month natural gas and heating oil futures returns. Next, significant temperature shock has effect on both the conditional mean and volatility of natural gas and heating oil prices. The results indicate that expected inventory surprises significantly and negatively affects the far-month natural gas futures returns. Moreover, volatility of natural gas futures returns is higher on Thursdays and that of near-month heating oil futures returns is higher on Wednesdays than other days. Finally, it is found that storage announcement for natural gas significantly affects near-month and far-month natural gas futures returns. Furthermore, both natural gas and heating oil futures returns are affected more by the weighted average temperature reported by multiple weather reporting stations than that reported by a single weather reporting station.

## 1. Introduction

During the past four decades, energy consumption has fluctuated markedly owing to fluctuation in energy demand and supply, as well as significant changes in climate conditions. Along with climate change and an increase in disasters frequency, global warming is a serious concern worldwide. Ruth et al. [1] argued that climate change, with associated events ranging from rising sea levels to strong and frequent storms and extreme temperature events, will significantly impact the natural environment and human infrastructure and its contribution to economic activity and quality of life. These impacts increase direct and indirect costs accrued from increasing environmental damage and disruption.

According to Chicago Mercantile Exchange (CME), the weather directly affects nearly 30% of the US economy. The US Energy Department estimated that $1 trillion of the US economy was exposed to weather risk in 2011. However, the notional value of traded weather derivatives was around US $3.5 billion, representing a mere fraction of total exposure. Consequently, weather derivatives are financial instruments provided by organizations or individuals to reduce or transfer risk associated with adverse or unexpected weather events. Organizations or individuals quantify weather in terms of how much temperature, frost, hurricane damage, or snowfall deviates from the monthly or seasonal average in a particular city or region. However, it is estimated that approximately 98.0% of currently traded weather derivatives are based on temperature. The first weather derivatives transaction was executed in the summer of 1997 by Aquila Energy as a weather option embedded in a power contract [2]. Gas, oil, and power companies use heating degree days (HDD) or cooling degree days (CDD) contracts to smooth earnings. HDD and CDD are among the most common weather derivatives.

This study examines and compares the impact of the temperature shock, expected natural gas and heating oil inventory surprises, movement of the Dow Jones industrial index (DJ), winter effect, storage announcement effect, hurricane announcement effect, and the nonlinear temperature effect on the conditional means and volatility of both near-month and far-month natural gas and heating oil futures returns.

## 2. Literature Review

Previous literatures are classified into two categories: one on the impact of temperature or weather on commodity futures; the other on natural gas and/or heating oil futures. Relevant studies in the first category include the following: Stevens [3] pointed out that the weather and climatology literature indicated persistence in North American weather patterns during the summer months. Given this nonrandom character of weather and given that the corn, wheat, and soybean belts are sufficiently geographically concentrated to be dominated by a regional weather phenomenon, their futures markets are hypothesized to reflect this assimilation of nonrandom weather information as nonrandom price fluctuations. Ates and Wang [4] found that extreme cold weather and inventory surprises influenced variation in basis, spot, and futures price changes. Furthermore, the conditional volatility of natural gas and heating oil spots and futures markets was higher in winter and lower in summer. Mu [5] examined how weather shocks influenced asset price dynamics in the US natural gas futures market. The empirical results revealed a significant weather effect on both the conditional mean and volatility of natural gas futures returns. Combined with the evidence that the volatility is significantly higher on Monday and on the day when the natural gas storage report is released, their findings suggested that information on market fundamentals significantly determines natural gas volatility. Chen et al. [6] examined the role of weather as a short-term demand factor and inventory as a short-term supply factor in explaining price spikes and time-varying volatility in natural gas spot and futures returns.

For studies in the second category, Herbert [7] summarized the relationship between spot and futures prices for natural gas, which could obtain accurate forecasts of spot prices. The natural gas futures market, thus, appeared inefficient. Walls [8] pointed out that the natural gas futures market was generally consistent with the efficient market hypothesis; that is, the futures market price was an unbiased predictor of spot prices at most market locations examined. Chinn et al. [9] examined the relationship between spot and futures prices for energy commodities (crude oil, gasoline, heating oil, and natural gas). Chinn et al. found that futures accurately predicted future spot prices, with the exception of 3-month natural gas futures. Chiou-Wei et al. [10] identified empirical regularities between changes in futures prices and surprise changes in natural gas in storage. Chiou-Wei et al. found an inverse relation between changes in futures prices and surprises in the change in natural gas in storage. Suenaga et al. [11] found that the volatility dynamics of NYMEX gas futures displayed two important features: (1) volatility is greater in winter than summer and (2) the persistence of price shocks and, hence, the correlation among currently traded contracts exhibited considerable seasonal and cross-sectional variation, consistent with the theory of storage. Recently, the usefulness of computational intelligence tools is highlighted by applying related models, such as neural networks and fuzzy sets, to natural gas consumption [12–14].

## 3. Methodologies

This study examines the influence of temperature change shock and inventory surprises on returns of near-month and far-month natural gas and heating oil futures returns from 2003 to 2006, extending the expected temperature and natural gas inventory shock model presented by Mu [5] to examine both natural gas and heating oil futures, and uses the Dow Jones index (DJ) provided by DataStream to proxy for equity market return. This investigation uses the daily temperature data from January 1, 2003 to December 31, 2006 provided by the NYMEX. Hurricane daily data is obtained from network of NYMEX and EQECAT. The other data sources are obtained from US Energy Information Administration and National Oceanic and Atmospheric Administration (NOAA). This investigation also examines whether the temperature reported by a single weather reporting station or the weighted average temperature reported by multiple weather reporting stations is more appropriate for examining the impact of temperature shock on energy futures returns. This study includes DJ returns, winter heating season, energy announcements, hurricane announcements, and nonlinear temperature as the independent parameters. As defined by contract 1 (namely, near-month) and contract 2 (namely, far-month) illustrated by EIA, contract 1 is a futures contract specifying the earliest delivery date; meanwhile, contract 2 represents the subsequent delivery month to that in contract 1.

To achieve the above objectives, the parameters are defined on natural gas and heating oil futures first; then the ADF test is applied to examine whether the parameters of the relevant models are stationary. Upon handling the stationary problem, this work examines whether the model is characterized by self-autocorrelations. If self-autocorrelation exists, the ARMA method is used to solve the self-autocorrelation problem of residuals. Furthermore, this study employs Ljung-Box Q^2^ and ARCH-LM methods to test for the ARCH effect. If the answer is positive, the GARCH model is used; if it is negative, then the ordinary least square (OLS) model is used.

### 3.1. Parameter Definition

#### 3.1.1. Rate of Return

The rate of return (ROR) on energy futures is calculated as follows:

(1) $$\begin{array}{c} {\text{RET}}_{Z,i,t}=\left({\ln Z}_{i,t}-{\ln Z}_{i,t-1}\right)\times 100, \end{array}$$

where *Z* represents natural gas closing price or heating oil closing price and *Z* = *N* suggests natural gas, while *Z* = *H* indicates heating oil; furthermore, RET_*Z*,*i*,*t*_ denotes natural gas or heating oil futures returns at day *t*; *i* = 1 suggests near-month futures, while *i* = 2 demonstrates far-month futures. ln⁡⁡*Z*_*i*,*t*_ and ln⁡⁡*Z*_*i*,*t*−1_ represent the nature log of the closing price of energy futures at day *t* and day *t* − 1, respectively.

#### 3.1.2. Expected Temperature Shock

The expected temperature shock is calculated on the basis of the models presented in [5]. However, this work uses the weather derivatives products traded on NYMEX. This investigation then divides the temperature of the weather reporting stations into the temperature reports for a single weather reporting station and the weighted average temperature for multiple weather reporting stations which are calculated from the four largest energy consumption states. The weights are as follows. (1) For natural gas: California, 0.28; New York, 0.27; Illinois, 0.25; and Michigan, 0.20; (2) for heating oil: New York, 0.30; Pennsylvania, 0.28; New Jersey, 0.23; and Massachusetts, 0.19. Expected temperature change shock for natural gas and heating oil is calculated as follows:

(2) $$\begin{array}{c} \;\;\;\;\;\;{\text{DD}}_{z,j,t}={\text{CDD}}_{z,j,t}+{\text{HDD}}_{z,j,t}\\ W_{z,j,t}=\frac{1}{n}\overset{n}{\underset{k=1}{\sum }}\left({\text{DD}}_{z,j,t+k}-{\text{ADDN}}_{z,j,t+k}\right), \end{array}$$

where DD_*z*,*j*,*t*_ denotes the sum of the cooling degree days (CDD) and heating degree days (HDD) of natural gas; *j* = 1 demonstrates the temperature reported by a single weather reporting station; *j* = 2 suggests the weighted average temperature reported by multiple weather reporting stations.  CDD_*z*,*j*,*t*_ demonstrates the CDD of natural gas at day *t*, which is the weighted average temperature reported by four weather reporting stations. Furthermore, HDD_*Z*,*j*,*t*_ is the HDD of energy futures at day *t*; *W*_*z*,*j*,*t*_ denotes the expected average temperature shock of energy futures at day *t*;  DD_*Z*,*j*,*t*+*k*_ is the sum of the CDD and the HDD for energy futures at day *t* + *k*; ADDN_*Z*,*j*,*t*+*k*_ represents the average degree days of energy futures at day *t* + *k* for the past 30 years; *n* = 7 denotes the weather forecast for change in weather conditions for the next 7 days.

#### 3.1.3. Expected Inventory Surprises

The change in expected inventory surprises for energy futures is calculated as follows:

(3) E(ΔIz,t)=c0+c1TZt+c2TZt2 +[λlsin⁡(2πw(t)52)+θlcos⁡⁡(2πw(t)52)]+μt,

where

(4) $$\begin{array}{c} {\;\mu }_{t}=\rho_{1}\mu_{t-1}+\eta_{t}+\eta_{t-1},\eta_{t}\sim N\left(0,1\right), \end{array}$$

where *E*(Δ*I*_*Z*,*t*_) denotes the change in market expectations regarding inventory surprises for energy futures from the Friday of week *t* − 1 to the Friday of week *t*; *TZ*_*t*_ represents the weighted weekly average temperature in week *t* of the energy futures; *w*(*t*) is a repeating step function in the Fourier series that cycles through 1,2,…52 (namely, each week of the year). Based on Schwarz information criterion (SIC), the number of lags is set in the Fourier and autoregressive series to test for a serial correlation. The change in expected inventory surprise for energy futures is defined as the difference between the announced storage change and the expected inventory change:

(5) EINVZτ=ΔIZ,τ−E(ΔIZ,τ)EINVZt=EINVZτ−1when  IDZt=1, IDZt=0,  otherwise,

where EINVZ_*τ*_ and EINVZ_*τ*−1_ denote the forecasting error of the weekly inventory for energy futures at weeks *τ* and *τ* − 1, respectively and extend weekly data into daily data. IDZ_*t*_ is a dummy variable of announced storage for energy futures. Since the weekly natural gas storage report is released by the EIA every Thursday, IDN_*t*_ = 1 for each Thursday, and IDN_*t*_ = 0 otherwise. On the other hand, the weekly heating oil reports released by the EIA use IDH_*t*_ = 1 for each Wednesday, and IDH_*t*_ = 0, otherwise.

#### 3.1.4. Dow Jones Industrial Index Return

We calculate the Dow Jones industrial index (DJ) returns as follows:

(6) $$\begin{array}{c} {\text{DJ}}_{t}=\left({\ln\text{DJ}}_{t}-{\ln\text{DJ}}_{t-1}\right)\times 100, \end{array}$$

where DJ_*t*_ denotes Dow Jones industrial index returns as day *t*. ln⁡⁡DJ_*t*_ and ln⁡⁡DJ_*t*−1_ represent the natural log of the closing prices of DJ on days *t* and *t* − 1.

#### 3.1.5. Winter Heating Season

We use the definition of EIA, with the winter heating season WIN_*t*_ running from October to March. The month lies between October and March if WIN_*t*_ = 1, whereas WIN_*t*_ = 0, otherwise.

#### 3.1.6. Hurricane Announcements (STM)

This investigation uses STM_*t*_ to represent hurricane announcements; the hurricane daily data^1^ is obtained from NYMEX. The sample period runs from Jan. 1, 2003 to Dec. 31, 2006. STM_*t*_ = 1 denotes that a hurricane's degree is 3 or higher. Otherwise, STM_*t*_ is equal to zero.

### 3.2. Considered Models

The following models are taken into account: (1) augmented Dickey-Fuller (ADF) test; (2) Ljung-Box test [15]; (3) autoregressive conditional heteroskedasticity (ARCH) test [16]; (4) ordinary least squares (OLS) test or generalized ARCH (GARCH) test. First, we use the augmented Dickey-Fuller (ADF) test [17, 18] to examine whether the series data is stationary. The error term in the Dickey-Fuller test is autocorrelated or the set of time series models is complicated. The Ljung-Box test is then used to examine whether any of the groups of autocorrelations of a time series are different from zero. If the Ljung-Box test shows that most parameters do not have autocorrelation problem, the autoregressive moving average (ARMA) model is not required. Gujarati [19] pointed out that, despite the large sample, both the Box-Pierce Q and the LB statistics follow the chi-square distribution. However, the LB statistic has better small-sample properties than the Q statistic. Third, the ARCH model is used to characterize and model observed time series. The key idea of the ARCH model is that the variance of the current error term depends on the actual size of the squared error term of the previous time.

The Lagrange multiplier test [20] is employed to test lag length for ARCH errors, namely, ARCH-LM test. Fourth, the GARCH model, a generalization of the ARCH model, is used to examine the influence of temperature on energy futures returns. A typical GARCH (*p*, *q*) model (where *p* denotes the order of the GARCH terms and *σ* ^2^ and *q* represent the order of the ARCH terms) is proposed by Bollerslev [16] and specified as follows:

(7) $$\begin{array}{c} {\;\;y}_{t}\;|\;\Omega_{t}\sim N\left(x_{t}a,\sigma_{t}^{2}\right)\\ {\;\;\;\varepsilon }_{t}=y_{t}-x_{t}a,\\ {\;\;\;\varepsilon }_{t}\;|\;\Omega_{t-1}\sim N\left(0,\sigma_{t}\right)\\ \sigma_{t}^{2}=\alpha_{0}+\overset{q}{\underset{i=1}{\sum }}\alpha_{i}\varepsilon_{t-i}^{2}+\overset{p}{\underset{j=1}{\sum }}\beta_{j}\sigma_{t-j}^{2}, \end{array}$$

where *x*_*t*_ denotes the independent variable vector; *a* represents the vector of the regression coefficient; *q* refers to the lag time; *x*_*t*_ *a* is the portfolio obtained from information set Ω_*t*_; *ε*_*t*_ represents the error term between the actual and the estimated values; *σ*_*t*_^2^ represents the conditional variance of time series 1/*t*. *σ*_*t*−*j*_^2^ denotes the conditional variance of return RET time series at day *t* − *j*, where *j* = 1 thru *P*.

The GARCH models is employed to test the mean and volatility of each studied parameter in relation to the influence of temperature on near-month and far-month natural gas and heating oil futures returns as follows:

(8) RETZ,i,t=a0+a1WZ,j,t+a2EINVz,t+a3DJt+εt  εt ∣ Ωt−1~N(0,σt2)σt2=α0α1εt−12+β1σt−12+b1WINt+b2IDZt +b3STMt+b4WZ,j,t+b5WZ,j,t2,

where *Z* represents natural gas or heating oil; *Z* = *N* suggests natural gas; *Z* = *H* indicates heating oil; RET_*Z*,*i*,*t*_ denotes the *i*th month return of natural gas or heating oil futures at day *t*; *i* = 1 suggests near-month futures; *i* = 2 indicates far-month futures. Ω_*t*−1_ denotes the total available information at day *t* − 1; *ε*_*t*−1_^2^ demonstrates the conditional variance is affected by the error term square at day *t* − 1; *W*_*Z*,*j*,*t*_ + *W*_*Z*,*j*,*t*_^2^ is the nonlinear effect of natural gas or heating oil at day *t* and *j*th temperature.

## 4. Empirical Results

**Figure 1:**
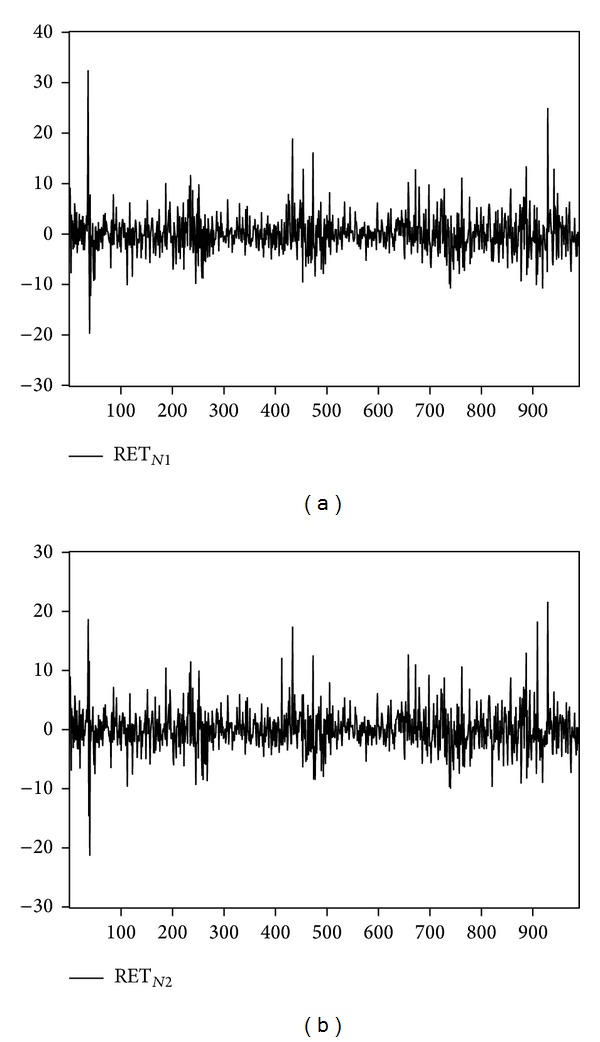
Return of natural gas: (a) near-month and (b) far-month.

**Figure 2:**
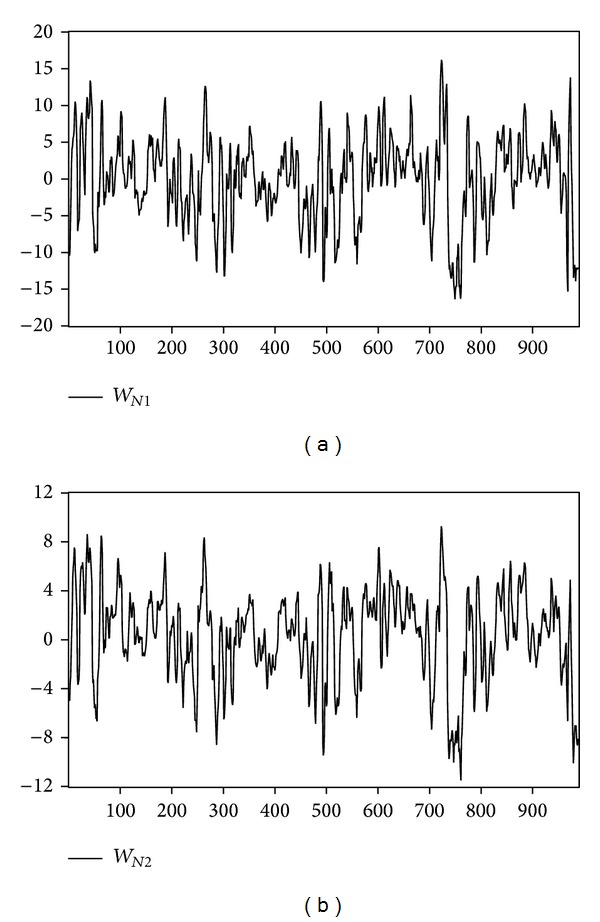
Temperature shock variable for natural gas.

**Figure 3:**
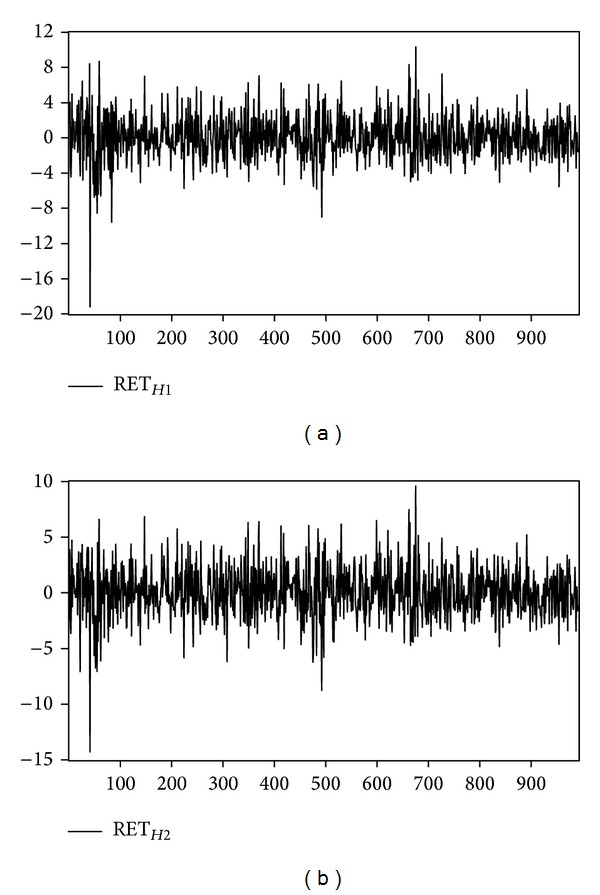
Returns of heating oil: (a) near-month and (b) far-month.

**Figure 4:**
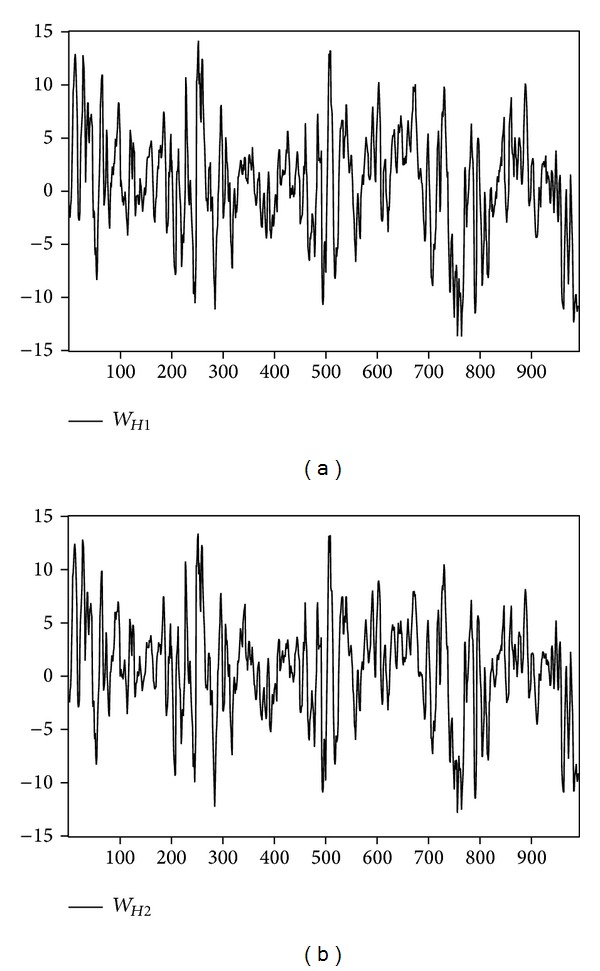
Temperature shock variable for heating oil.

We examine the influence of temperature and inventory change shocks on natural gas and heating oil futures returns during 2003 to 2006. Upon deleting the missing data for any parameter, there are 988 original data samples for natural gas, and 991 for heating oil.  Figure 1 depicts the returns of near-month and far-month natural gas futures.  Figure 2 depicts the trend of temperature shock variable for natural gas for one weather reporting station (i.e., Chicago) and multiple weather reporting stations (i.e., California, New York, Illinois, and Michigan).  Figure 3 shows the returns of heating oil for near-month and far-month futures. As for Figure 4, it depicts the trends of temperature shock variables for heating oil for a single weather reporting station (i.e., New York) and multiple weather reporting stations (i.e., New York, Pennsylvania, New Jersey, and Massachusetts).

Table 1 shows that each parameter in a time series sample is stationary. Ljung-Box *Q* tests are then employed to examine autocorrelation for all the natural gas and heating oil series. In Table 2, series 1 denotes energy futures returns are affected by the temperature reported by a single weather reporting, and series 2 denotes energy futures returns are affected by the weighted average temperature reported by four weather stations. *Q*(*k*) is the Ljung-Box statistic value of *k*-level time lag of returns series. Table 2 indicates that, under the Ljung-Box tests, most variables of series 1 and 2 for natural gas and heating oil futures are insignificant, suggesting that natural gas and heating oil futures are nearly free of autocorrelation. The autoregressive moving average (ARMA) model, thus, is not required.

Table 3 shows that both Ljung-Box *Q* ^2^ and ARCH-LM statistics are significant, suggesting heteroskedasticity. This study, thus, uses the GARCH model rather than the OLS model to estimate and analyze the influence of temperature change shock and inventory surprises on natural gas and heating oil futures returns.  Table 4 lists the optimum GARCH results for the influence of the expected temperature shock based on the temperature reported by a single weather reporting station or the weighted average temperature reported by multiple weather reporting stations on energy futures returns. These findings indicate that the log likelihood values from the weighted average temperature reported by multiple weather reporting stations are lower than those from a single weather reporting station for energy futures returns, suggesting that the average weighted temperature data obtained from multiple weather reporting stations are more appropriate for examining the impact of temperature change on energy futures returns than those obtained from a single weather station. That means the average weighted temperature should be used to estimate the GARCH model for energy futures returns. Empirical results show that W_N1_, W_N2_, W_H1_, and W_H2_ are all significantly positive for both near-month and far-month natural gas and heating oil futures returns, suggesting that when the market expects the degree day in the future will be higher (lower) than the average degree day level, the energy demand will increase (decrease), causing near-month and far-month natural gas or heating oil futures returns to increase (decrease).  Table 4 also shows that expected inventory surprises of heating oil do not significantly impact heating oil futures returns. However, this study finds that the expected inventory surprises of natural gas are significantly and negatively related with natural gas futures returns, which is consistent with the findings of Ates and Wang [4].  Table 4 also shows that DJ is significantly and negatively associated with heating oil futures returns suggesting that the increase (decrease) of DJ decreases (increases) near-month and far-month heating oil futures returns.

Temperature in Table 4 is reported by single and multiple weather reporting stations. Regarding the variance equations, this study finds data for the winter heating season, as listed in Table 4, is mostly insignificant, suggesting that energy futures returns are generally not higher during the winter than other seasons, which is inconsistent with the findings in [5]. The adjusted *R*-squares range for the temperature reported by one weather reporting station is from 0.003 to 0.027; that range for the temperature reported by multiple weather reporting stations is 0.005 to 0.026. Empirical results show that both natural gas and heating oil's announcement day is significantly positive, suggesting that a storage report announcement effect exists, which is consistent with the literature. This work finds that hurricane announcement effect does not exist for energy futures returns.

This investigation also finds significantly positive nonlinear temperature effects for natural gas futures returns, suggesting the market expects degree day volatility to increase with natural gas demand. Furthermore, since the sum of GARCH coefficients can measure the persistence of market volatility, this study finds that both the sum of the coefficients affected by the temperature reported from a single weather reporting station and that from weighted average temperature of multiple weather reporting stations approaches one, except for the far-month natural gas futures prices, consistent with the stable convergence of variance in the GARCH model.

## 5. Conclusion

We used the GARCH model to examine the influence of the unexpected temperature shock based on both the temperature reported by a single weather reporting station and the weighted average temperature reported by multiple weather reporting stations, expected inventory surprise, the movement of Dow Jones Industrial Index (DJ), winter heating season, storage announcements, hurricane announcements, and nonlinear effect of temperature on near-month and far-month energy natural gas and heating oil futures returns. Several interesting results are summarized as follows.

1. The expected temperature shock significantly and positively affects near-month and far-month natural gas and heating oil futures returns. The implication is that, along with the drastic change in temperatures over past four decades, the uncertainly of economic environmental factors increases, increasing the volatility of natural gas and heating oil prices and raising the required rate of returns on natural and heating oil futures. This suggests that the investors should invest a portion of their funds on the weather derivative commodities for their hedging and arbitraging purposes.
2. Significant temperature effect on both the conditional mean and volatility of both natural gas and heating oil futures prices, suggesting that when the market expects the degree days in the future to be higher than average, energy demand increase, increasing the near-month and far-month natural gas and heating oil futures returns. This result confirms with the finding of Mu [5] for natural gas futures.
3. Expected inventory surprises significantly and negatively impacts the far-month natural gas futures returns, confirming the finding in [10, 21]. However, the expected inventory surprises do not affect heating oil futures returns.
4. Expected nonlinear temperature effect exists for natural gas futures returns, suggesting the market expects the degree day volatility to increase with natural gas demand. However, nonlinear temperature effects does not exist for heating oil futures returns.
5. DJ returns significantly and negatively affect heating oil futures returns.
6. Storage announcement for natural gas significantly affects near-month and far-month natural gas futures returns; while storage announcements for heating oil significantly affect near-month heating oil futures returns. These results show that information about market fundamentals is an important determination of near-month energy futures returns.
7. The hurricane announcement and winter heating season do not significantly affect natural gas and heating oil futures returns; the latter is inconsistent with the findings in [4, 5].
8. Using the GARCH, the results indicate that the weighted average temperature data obtained from multiple weather reporting stations is more appropriate than that got from a single weather reporting station for examining the impact of the temperature shock on natural gas and heating oil futures returns.

## 6. Limitations and Future Research Directions

This study has the following restrictions.

1. The temperature data of US weather stations at each state is difficult for collection, causing the sample period of this study to be limited to four years.
2. Since a large portion of the near-month and the far-month's energy products data are hard to collect, the research targets of this study regarding energy commodities are limited to natural gas and heating oil only.
3. Since most of the previous researches examined the impact of the temperature on the agricultural commodity futures, only a few studies examined that on the energy product futures; the relevant references for this investigation are limited.

This investigation suggests future researchers to examine the impact of temperature on other energy commodities in addition to the natural gas and heating oil or to include longer sample period than four years for various energy products futures returns.

## Figures and Tables

**(a):** 

Natural gas model	ADF statistics-original series			PP statistics-original series		
Variables	Lag term	w/constant w/o time trend	w/constant & w/time trend	Lag term	w/constant w/o time trend	w/constant & w/time trend

RET_*N*1_	0	−31.7562∗∗∗	−31.7437∗∗∗	4	−31.7534∗∗∗	−31.7411∗∗∗
RET_*N*2_	0	−33.1140∗∗∗	−33.1049∗∗∗	1	−33.1135∗∗∗	−33.1043∗∗∗
*W*_*N*1_	6	−7.3796∗∗∗	−7.3917∗∗∗	37	−5.7894∗∗∗	−5.8117∗∗∗
*W*_*N*2_	9	−5.9611∗∗∗	−6.0443∗∗∗	21	−5.6500∗∗∗	−5.7281∗∗∗
EINVN	5	−6.0680∗∗∗	−6.3519∗∗∗	17	−7.4740∗∗∗	−7.7667∗∗∗
DJ	0	−33.7349∗∗∗	−33.7173∗∗∗	1	−33.7367∗∗∗	−33.7191∗∗∗

**(b):** 

Heating oil model	ADF statistics-original series			PP statistics-original series		
Variables	Lag term	w/constant w/o time trend	w/constant & w/time trend	Lag term	w/constant w/o time trend	w/constant & w/time trend

RET_*H*1_	0	−33.4204∗∗∗	−33.4068∗∗∗	7	−33.4627∗∗∗	−33.4494∗∗∗
RET_*H*2_	0	−33.4655∗∗∗	−33.4534∗∗∗	9	−33.5213∗∗∗	−33.5103∗∗∗
*W*_*H*1_	6	−7.5365∗∗∗	−7.8002∗∗∗	26	−5.3673∗∗∗	−5.5103∗∗∗
*W*_*H*2_	6	−8.0559∗∗∗	−8.3002∗∗∗	26	−5.4189∗∗∗	−5.5319∗∗∗
EINVH	5	−6.5395∗∗∗	−6.5298∗∗∗	10	−7.1929∗∗∗	−7.1846∗∗∗
DJ	0	−33.7270∗∗∗	−33.7095∗∗∗	1	−33.7282∗∗∗	−33.7107∗∗∗

***1% significance level; **5% significance level; *10% significance level; RET_*N*1_: natural gas near-month futures return; RET_*N*2_: natural gas far-month futures return; RET_*H*1_: heating oil near-month futures return; RET_*H*2_: heating oil far-month futures return; *W*_*N*1_: expected temperature shock for natural gas reported by single weather reporting station; *W*_*N*2_: expected weighted average temperature shock for natural gas reported by four weather reporting stations; *W*_*H*1_: expected temperature shock for heating oil reported by one weather reporting station; *W*_*H*2_: expected weighted average temperature shock for heating oil reported by four weather reporting stations; EINVN: expected inventory shock for natural gas; EINVH: expected inventory shock for heating oil; DJ: Dow Jones industrial index.

**Table 2:** Results for Ljung-Box *Q* test.

Commodity	Variable	*Q*(1)	*Q*(2)	*Q*(3)	*Q*(4)	*Q*(5)	*Q*(6)	*Q*(9)	*Q*(12)
RET_*N*1_	Series 1	0.1670	0.3630	1.8291	2.2173	4.1915	4.6245	6.7521	8.0710
		(0.6830)	(0.8340)	(0.6090)	(0.6960)	(0.5220)	(0.5930)	(0.6630)	(0.7800)
	Series 2	0.2228	0.3636	2.0589	2.3091	4.5976	5.1488	7.2407	8.6039
		(0.6370)	(0.8340)	(0.5600)	(0.6790)	(0.4670)	(0.5250)	(0.6120)	(0.7360)

RET_*N*2_	Series 1	2.9185	3.9548	4.2424	4.2924	5.4078	5.6102	7.7109	11.3040
		(0.0880∗)	(0.1380)	(0.2360)	(0.3680)	(0.3680)	(0.4680)	(0.5640)	(0.5030)
	Series 2	3.1149	4.0195	4.3823	4.4941	5.8186	6.0655	8.1283	11.5900
		(0.0780∗)	(0.1340)	(0.2230)	(0.3430)	(0.3240)	(0.4160)	(0.5210)	(0.4790)

RET_*H*1_	Series 1	3.9518	4.5093	4.5625	6.0989	6.1009	6.6292	8.5532	10.4580
		(0.0470∗∗)	(0.1050)	(0.2070)	(0.1920)	(0.2970)	(0.3570)	(0.4790)	(0.5760)
	Series 2	3.9350	4.4883	4.5397	6.0974	6.0986	6.6583	8.5968	10.4910
		(0.0470∗∗)	(0.1060)	(0.2090)	(0.1920)	(0.2970)	(0.3540)	(0.4750)	(0.5730)

RET_*H*2_	Series 1	3.8889	4.1075	4.2889	5.7237	6.0139	6.7367	8.1229	9.7870
		(0.0490∗∗)	(0.1280)	(0.2320)	(0.2210)	(0.3050)	(0.3460)	(0.5220)	(0.6350)
	Series 2	3.8486	4.0742	4.2522	5.7043	6.0079	6.7678	8.1760	9.8194
		(0.0500*)	(0.1300)	(0.2350)	(0.2220)	(0.3050)	(0.3430)	(0.5170)	(0.6320)

**Table 3:** Results for Ljung-Box *Q* ^2^ and ARCH-LM tests.

Commodity	Variables	*Q* ^2^(3)	*Q* ^2^(6)	*Q* ^2^(9)	*Q* ^2^(12)	ARCH(6)	ARCH(12)
RET_*N*1_	Series 1	37.4260	47.8960	48.1930	57.5900	7.4251	4.3999
		(0.0000∗∗∗)	(0.0000∗∗∗)	(0.0000∗∗∗)	(0.0000∗∗∗)	(0.0000∗∗∗)	(0.0000∗∗∗)
	Series 2	37.7920	47.9380	48.2280	57.8320	7.4365	4.4098
		(0.0000∗∗∗)	(0.0000∗∗∗)	(0.0000∗∗∗)	(0.0000∗∗∗)	(0.0000∗∗∗)	(0.0000∗∗∗)

RET_*N*2_	Series 1	49.2860	49.7660	50.2800	58.3290	7.0381	4.4487
		(0.0000∗∗∗)	(0.0000∗∗∗)	(0.0000∗∗∗)	(0.0000∗∗∗)	(0.0000∗∗∗)	(0.0000∗∗∗)
	Series 2	48.7730	49.2380	49.7220	57.6940	6.9851	4.4071
		(0.0000∗∗∗)	(0.0000∗∗∗)	(0.0000∗∗∗)	(0.0000∗∗∗)	(0.0000∗∗∗)	(0.0000∗∗∗)

RET_*H*1_	Series 1	9.4883	22.1670	29.4190	33.8710	3.1697	2.1361
		(0.0230∗∗)	(0.0010∗∗∗)	(0.0010∗∗∗)	(0.0010∗∗∗)	(0.0043∗∗∗)	(0.0129∗∗)
	Series 2	9.5893	22.1060	29.3000	33.7370	3.1614	2.1273
		(0.0220∗∗)	(0.0010∗∗∗)	(0.0010∗∗∗)	(0.0010∗∗∗)	(0.0044∗∗∗)	(0.0133∗∗)

RET_*H*2_	Series 1	1.2064	13.6810	22.5980	28.2530	2.3223	2.1579
		(0.7510)	(0.0330∗∗)	(0.0070∗∗∗)	(0.0050∗∗∗)	(0.0311∗∗)	(0.0118∗∗)
	Series 2	1.2168	13.4810	22.4570	28.1050	2.2882	2.1480
		(0.7490)	(0.0360∗∗)	(0.0080∗∗∗)	(0.0050∗∗∗)	(0.0336∗∗)	(0.0123∗∗)

**Table 4:** Summary of the near-month and far-month energy futures prices.

RET_*Z*1_ − *W*_*Z*1_	A single weather station				RET_*Z*2_ − *W*_*Z*2_	Multiple weather stations			
Mean Eqn	NN	FN	NH	FH	Mean Eqn	NN	FN	NH	FH
Variables	Coefficients (*P* value)				Variables	Coefficients (*P* value)			
*C*	0.1464	0.1595	0.0921	0.0933	*C*	0.1060	0.1382	0.8816	0.0914
(*P* value)	(−0.2258)	(0.1416)	(0.2187)	(0.1938)	(*P* value)	(0.3851)	(0.2324)	(0.2406)	(0.2046)

*W*_*Z*1_	0.062∗∗∗	0.0488∗∗∗	0.0315∗∗	0.0093∗∗	*W*_*Z*2_	0.1038∗∗∗	0.0983∗∗∗	0.0310∗	0.0287∗
(*P* value)	(−0.0037)	(0.0027)	(0.0389)	(0.0442)	(*P* value)	(0.0017)	(0.0020)	(0.0583)	(0.0682)

EINVZ	−0.0095∗	−0.0141∗∗	−0.0001	0.0000	EINVZ	−0.0095∗	−0.0104∗∗	−0.0001	0.0000
(*P* value)	−0.0656	(0.0435)	(0.1354)	(0.2134)	(*P* value)	(0.0738)	(0.0397)	(0.1551)	(0.2390)

DJ	−0.2378∗	−0.1876	−0.3564∗∗∗	−0.4307∗∗∗	DJ	−0.2436	−0.1586	−0.3627∗∗∗	−0.4365∗∗∗
(*P* value)	(−0.0985)	(0.2714)	(0.0007)	(0.0000)	(*P* value)	(0.1020)	(0.2667)	(0.0004)	(0.0000)

Variance Eqn	Coefficients (*P* value)				Variance Eqn	Coefficients (*P* value)			

*C*	0.2375	0.541∗∗∗	−0.0395	0.0349	*C*	0.1262	2.8616∗∗∗	−0.0822	0.0000
(*P* value)	(−0.5209)	(0.0048)	(0.8130)	(0.8535)	(*P* value)	(0.7076)	(0.0025)	(0.6111)	(0.9994)

ARCH(1)	0.1273∗∗∗	0.0230∗∗∗	0.0460∗∗∗	0.0246∗∗	ARCH(1)	0.1071∗∗∗	0.1288∗∗∗	0.0431∗∗∗	0.0247∗∗
(*P* value)	(0.0000)	(0.0000)	(0.0020)	(0.0244)	(*P* value)	(0.0000)	(0.0000)	(0.0028)	(0.0196)

GARCH(1)	0.7956∗∗∗	0.9669∗∗∗	0.9026∗∗∗	0.9250∗∗∗	GARCH(1)	0.8321∗∗∗	0.5425∗∗∗	0.9092∗∗∗	0.9277∗∗∗
(*P* value)	(0.0000)	(0.0000)	(0.0000)	(0.0000)	(*P* value)	(0.0000)	(0.0000)	(0.0000)	(0.0000)

WIN	0.1474	−0.1791	0.1671∗	0.1348∗	WIN	0.1495	−0.2403	0.1415	0.1165
(*P* value)	(−0.4743)	(0.4894)	(0.0535)	(0.0723)	(*P* value)	(0.3728)	(0.4829)	(0.1033)	(0.1029)

IDZ	2.6683∗∗	−2.1726∗∗∗	1.3818∗	0.8908	IDZ	2.1381∗	2.7170∗∗∗	1.4443∗∗	0.9592
(*P* value)	(−0.0329)	(0.0011)	(0.0577)	(0.1852)	(*P* value)	(0.0839)	(0.0006)	(0.0446)	(0.1491)

STM	1.0145	0.7189	−0.0752	−0.016	STM	1.0828	1.7002	−0.0616	−0.0755
(*P* value)	(−0.1658)	(0.1017)	(0.6786)	(0.4876)	(*P* value)	(0.1036)	(0.1036)	(0.7282)	(0.5659)

*W*_*Z*1_	0.1108∗∗∗	0.0068∗∗	0.0246∗∗	0.0202∗∗	*W*_*Z*2_	0.1186∗∗∗	0.1296∗∗	0.0278∗∗∗	0.0227∗∗
(*P* value)	(0.0000)	(0.0164)	(0.0053)	(0.0145)	(*P* value)	(0.0000)	(0.0272)	(0.0026)	(0.0103)

*W*_*Z*1_^2^	0.0102∗∗∗	0.0013∗∗∗	−0.0008	−0.0011	*W*_*Z*2_^2^	0.0169∗∗∗	0.0381∗∗∗	−0.0003	−0.0007
(*P* value)	(−0.0011)	(0.0021)	(0.5871)	(0.3258)	(*P* value)	(0.0031)	(0.0017)	(0.8652)	(0.6057)

Sum of coeff	0.9229	0.9899	0.9486	0.9496	Sum of coeff	0.9391	0.6713	0.9524	0.9524

Log likelihood	−2661.77	−2591.84	−2273.66	−2207.37	Log likelihood	−2662.70	−2604.80	−2273.78	−2207.65

***1% significance level; **5% significance level; *10% significance level.

NN: near-month natural gas; NH: near-month heating oil; FN: far-month natural gas; FH: far-month heating oil.
